# Attendance rate in icu follow up clinics: an analysis of patients that attend the icu clinic compared to those that do not attend

**DOI:** 10.1186/2197-425X-3-S1-A767

**Published:** 2015-10-01

**Authors:** M Colomo, E Smithers, J Cherian, A Pareed, G De la Cerda

**Affiliations:** Queen's Hospital, London, United Kingdom

## Introduction

In the UK, the implementation of ICU follow up services is becoming a standard of care after the National Institute of Health and Care Excellence (NICE) in 2009 published clinical guideline 83 (CG83) on rehabilitation after critical illness[1].

## Objectives

To compare the characteristics of the patients that attend our ICU follow up clinic with those that do not attend (DNA) with the objective of improving the selection of patients and decreasing the DNA rate.

## Methods

Retrospective cohort analysis of prospective collected data at a tertiary centre from November 2011 to March 2015. The clinic is run monthly by an ICU consultant and a critical care outreach nurse. Criteria to be invited to the clinic are mechanical ventilation ≥ 3 days. Demographics, duration of mechanical ventilation, ICU and hospital length of stay, attendance/DNA to the follow up services and reason for not attendance were collected.

## Results

A total of 116 patients met the criteria to be invited to the clinic. DNA rate was 44% which is similar to other series reported in the literature. Patients who DNA required a longer length of mechanical ventilation (10.05 days vs 12.42; p = 0.24), and a longer LOS in ICU (16.11 VS 19.96; p = 0.13) and in hospital (30.7 vs 34.5; p = 0.31) although these differences are not statistically significant. Regarding the known reasons for not attending clinic, (in 39% of the cases the reason was unknown) the most frequent one was that the patient was very well and did not feel beneficial to attend (12 patients). On the contrary, the rest of the patients who failed to attend were either too unwell & disabled (6), still in hospital & readmitted (9), had no transport means (5), or had died (4).

## Conclusions

1. The DNA rate in our clinic is still high.
2. Patients that do not attend are either too well or too sick to attend.
3. It is necessary to improve our selection criteria to this clinic to decrease the DNA rate and optimize the service.

**Figure 1:**
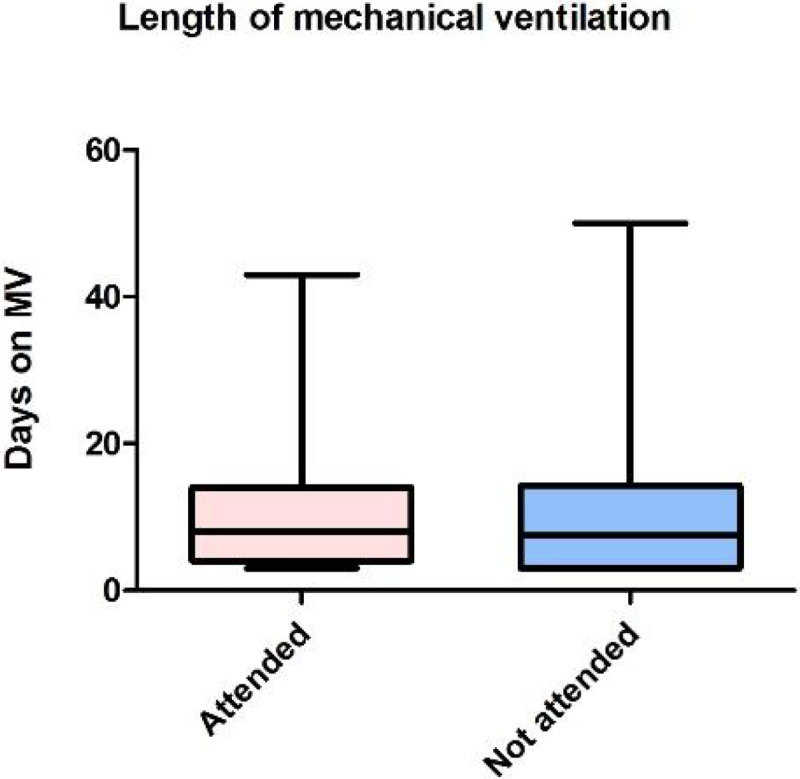
**Length of MV.**

**Figure 2:**
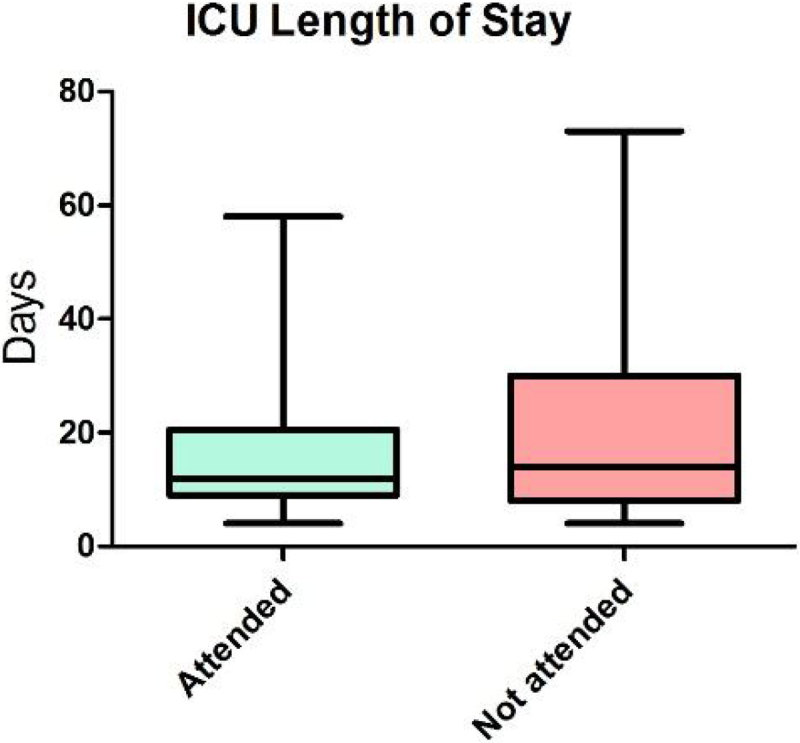
**ICU LOS.**

**Figure 3:**
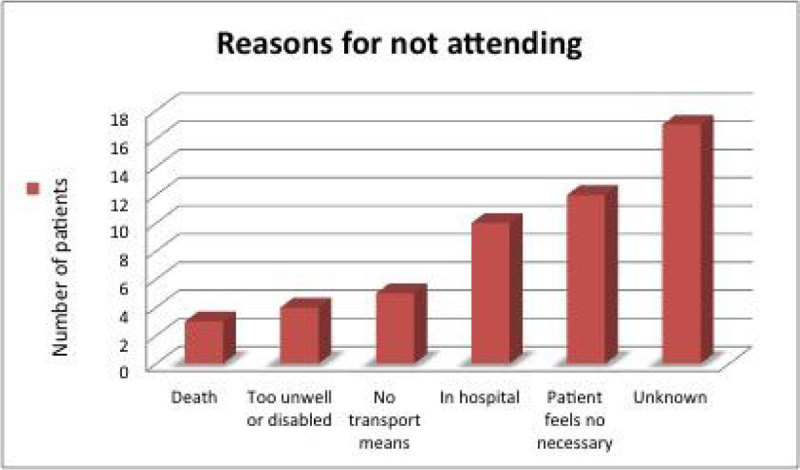
**Reasons for not attending.**
